# Using Satellites to Track Indicators of Global Air Pollution and Climate Change Impacts: Lessons Learned From a NASA‐Supported Science‐Stakeholder Collaborative

**DOI:** 10.1029/2020GH000270

**Published:** 2020-07-01

**Authors:** Susan C. Anenberg, Matilyn Bindl, Michael Brauer, Juan J. Castillo, Sandra Cavalieri, Bryan N. Duncan, Arlene M. Fiore, Richard Fuller, Daniel L. Goldberg, Daven K. Henze, Jeremy Hess, Tracey Holloway, Peter James, Xiaomeng Jin, Iyad Kheirbek, Patrick L. Kinney, Yang Liu, Arash Mohegh, Jonathan Patz, Marcia P. Jimenez, Ananya Roy, Daniel Tong, Katy Walker, Nick Watts, J. Jason West

**Affiliations:** ^1^ Milken Institute School of Public Health George Washington University Washington DC USA; ^2^ Nelson Institute Center for Sustainability and the Global Environment University of Wisconsin Madison WI USA; ^3^ School of Population and Public Health The University of British Columbia Vancouver British Columbia Canada; ^4^ Institute for Health Metrics and Evaluation University of Washington Seattle WA USA; ^5^ Clean Air Institute Washington DC USA; ^6^ Now at Pan‐American Health Organization Washington DC USA; ^7^ Climate and Clean Air Coalition to Reduce Short‐Lived Climate Pollutants Washington DC USA; ^8^ NASA Goddard Space Flight Center Greenbelt MD USA; ^9^ Lamont‐Doherty Earth Observatory Columbia University Palisades NY USA; ^10^ Pure Earth New York NY USA; ^11^ College of Engineering and Applied Science University of Colorado Boulder Boulder CO USA; ^12^ Department of Environmental and Occupational Health Sciences University of Washington Seattle WA USA; ^13^ James T.H. Chan School of Public Health Harvard T.H. Chan School of Public Health Boston MA USA; ^14^ C40 Cities New York NY USA; ^15^ School of Public Health Boston University School of Public Health Boston MA USA; ^16^ Rollins School of Public Health Emory University Atlanta GA USA; ^17^ Environmental Defense Fund Washington DC USA; ^18^ Center for Spatial Science and Systems George Mason University Fairfax VA USA; ^19^ Health Effects Institute Boston MA USA; ^20^ Lancet Countdown University College London London UK; ^21^ Gillings School of Global Public Health University of North Carolina at Chapel Hill Chapel Hill NC USA

**Keywords:** satellite remote sensing, air pollution, climate change, environmental surveillance, public health surveillance

## Abstract

The 2018 NASA Health and Air Quality Applied Science Team (HAQAST) “Indicators” Tiger Team collaboration between NASA‐supported scientists and civil society stakeholders aimed to develop satellite‐derived global air pollution and climate indicators. This Commentary shares our experience and lessons learned. Together, the team developed methods to track wildfires, dust storms, pollen counts, urban green space, nitrogen dioxide concentrations and asthma burdens, tropospheric ozone concentrations, and urban particulate matter mortality. Participatory knowledge production can lead to more actionable information but requires time, flexibility, and continuous engagement. Ground measurements are still needed for ground truthing, and sustained collaboration over time remains a challenge.

## Key Points

- The NASA Health and Air Quality Applied Science Team “Indicators” Tiger Team developed satellite‐based air quality and climate indicators
- Participatory knowledge production can lead to more useful information for stakeholders but requires continuous engagement and flexibility
- Ground measurements are still needed, and sustained collaboration between the researchers and stakeholders over time remains a challenge

## 1 Introduction

Several large‐scale and high‐profile efforts have recently highlighted the need for indicators measuring the world's progress toward mitigating health impacts of pollution and climate change. These efforts include the Lancet Commission on Pollution and Health (Landrigan et al., 2017), the Lancet Countdown: Tracking Progress on Health and Climate Change (Watts et al., 2019), the Global Burden of Disease (GBD) Study (GBD 2017 Risk Factor Collaborators, 2018), and State of the Global Air (Health Effects Institute and Institute for Health Metrics and Evaluation, 2019), all of which have plans for regular updating (in some cases, annually). Each iteration of these efforts expands and builds upon the data and indicators used for tracking pollution and, in the case of the Lancet Countdown, the links between health and climate change. By tracking changes observed for a set of well‐defined indicators over time, these reports can provide insights on both progress from prior policy decisions and the need for additional mitigation. Incorporating new exposures and indicators also improves the validity of the studies' estimates.

As these global‐scale public health modeling collaboratives have matured, the public health community increasingly relies on remote sensing for understanding air pollution exposures, including both abundances and spatiotemporal patterns. A main challenge for conducting global‐scale air quality and climate surveillance is the need for high‐quality observational data with global coverage, high spatial resolution, and standardized, continuous observations over long time periods. Ground monitors are sparsely located, even in countries with extensive monitoring networks, and remain nonexistent throughout much of the world (Martin et al., 2019). However, recent advances in satellite remote sensing now enable observation‐based tracking of climate and air pollution with relatively high spatial resolution everywhere on Earth. Remote sensing measurements are also consistent across time and space, whereas ground monitoring protocols and instruments change over time and are not harmonized across countries.

With these advantages, satellite remote sensing is disrupting the traditional paradigm of tracking air pollution concentrations and associated health risks that has relied on ground‐based networks of air quality monitoring stations. Important limitations remain, such as in algorithms used to derive surface conditions from atmospheric column measurements. However, recent and forthcoming advances are rapidly narrowing these uncertainties. Some satellite‐based sensors have now built long observational records (e.g., the Multi‐Angle Implementation of Atmospheric Correction [MAIAC] Land Aerosol Optical Depth [AOD] since 2000 and NO_2_ columns from the Ozone Monitoring Instrument [OMI] since 2005), while others launched more recently are improving the temporal frequency, geographic coverage, and spatial resolution of observations (e.g., the TROPOspheric Monitoring Instrument [TROPOMI] in operation since 2017 and the Geostationary Environment Monitoring Spectrometer [GEMS] since February 2020; Kim et al., 2020). We expect further expansion of satellite capabilities for air pollution and climate tracking through forthcoming launches focused specifically on air quality (e.g., Tropospheric Emissions: Monitoring Pollution [TEMPO], Zoogman et al., 2017, and the Multi‐Angle Imager for Aerosols [MAIA], Diner et al., 2018, both scheduled for 2022).

While the scope and scale of spaceborne Earth observations increasingly matches the needs of global health assessments, developing, analyzing, and interpreting these data sets for environmental and public health surveillance require technical expertise. This gap between production and use of scientific information has been well documented in other environmental decision‐making contexts (Kirchhoff et al., 2013; Norström et al., 2020) and is not unique to the application of satellite remote sensing for tracking global air quality and climate change. Bridging knowledge production to use requires new, problem‐driven, and participatory approaches to generating scientific information with mutual understanding and collaboration between scientists and end users. A key challenge is that scientists lending their expertise to produce and interpret relevant data sets often do so on a volunteer basis, limiting their ability to devote the time and effort needed to transition scientific research into actionable environmental and public health knowledge.

In 2018, the NASA Health and Air Quality Applied Science Team (HAQAST, pronounced “hay‐kast”) launched a new “Tiger Team” aimed at facilitating direct engagement of HAQAST researchers in global pollution and climate change surveillance reports. HAQAST, supported by NASA from 2016 to 2020, represents an innovative model of research funding to connect NASA's Earth science data with user needs in the air quality management and public health communities (Holloway et al., 2018). HAQAST Tiger Teams are short‐term, high‐impact collaborative efforts between HAQAST members and stakeholders to identify and solve an immediate problem using NASA data and products. The “Indicators” Tiger Team in particular initiated a new collaboration between HAQAST members and key civil society stakeholders with the goal of developing satellite‐derived global air pollution and climate indicators. Notably, it was one of the first projects supported by the NASA Applied Sciences Division to carry out applied research on air pollution and climate change at the global scale.

The purpose of this Commentary is to demonstrate the broad range of air quality and climate change tracking uses for satellite remote sensing data, share the experience of the NASA HAQAST Indicators Tiger Team, and relay lessons learned for harnessing the strengths of diverse teams to address complex societal challenges.

## 2 Methods

The Indicators Tiger Team launched in October 2018 with three main stakeholder organizations: the Lancet Commission on Pollution and Health (which later launched the Global Pollution Observatory), the Lancet Countdown on Health and Climate Change, and the Health Effects Institute, which publishes the State of Global Air reports and website. The team included ~20 researchers who were sponsored by NASA as part of HAQAST and aimed to produce six global air quality or climate change indicators that were of particular interest to the stakeholder organizations on the team: tropospheric ozone concentrations, nitrogen dioxide (NO_2_) concentrations, air pollution‐attributable premature mortality in cities worldwide, direct population exposure to wildfires, pollen season start date and duration, and dust storm frequency. During the course of the year‐long project, the team evolved to respond to emerging ideas and needs. New stakeholders joined the team, including the GBD Study, the Climate and Clean Air Coalition, C40 Cities, Clean Air Institute, and Environmental Defense Fund. In an effort to maximize the impact of our research, a two‐way learning process was built into the heart of this project. In some cases, researchers were “embedded” within the stakeholder organizations from the beginning, which helped ensure the indicators produced were policy and stakeholder relevant. In others, the two‐way learning process entailed researchers presenting on their methods and capabilities and stakeholders presenting on their objectives and needs, which then sparked smaller spin‐off discussions where researchers and stakeholders narrowed in on collaborative opportunities to develop satellite‐based indicators that could be integrated into the stakeholders' activities. As a result, the team added several new indicator topics: NO_2_‐attributable asthma burdens, NO_2_ changes from urban vehicle policies, and a measure of urban green space. The team concluded in summer 2020, though some work continues with support from HAQAST and other sources.

Here we briefly summarize the Tiger Team's work for each of the air quality and climate change indicators. The indicators are grouped by whether they are primarily used as a climate change indicator or as an air quality indicator, though air pollution and climate change are highly interrelated (e.g., wildfire occurrence is used here as an indicator of climate change and also indicates poor air quality due to smoke exposure). For each topic, we describe the motivation for addressing the topic through the Tiger Team, methods used to leverage satellites or information from other geoscientific sources (e.g., chemical transport models), interactions between the NASA‐funded researchers and stakeholders, and any outcomes completed or planned. This Commentary complements more detailed technical articles for each indicator; thus, methods are only briefly summarized here. We did not seek to address development of global and country scale PM_2.5_ concentrations, as such data sets are already well established and widely used, even though uncertainties remain (Diao et al., 2019).

### 2.1 Indicators Tracking Effects of Climate Change and Associated Mitigation Efforts

#### 2.1.1 Wildfire Exposure

The higher temperature, changing precipitation patterns, and decreasing soil moisture related to climate change increase the likelihood of wildfires in many parts of the world (Liu et al., 2010). In addition to property damage and firefighting expenses, wildfires have substantial adverse health impacts either directly from burns, injuries, and mortality or indirectly via smoke inhalation on morbidity and mortality from acute and chronic respiratory and cardiovascular outcomes (Black et al., 2017). Although wildfires are projected to worsen with climate change, no global indicators currently exist to track direct population exposures to wildfires and their long‐term trends. We collaborated with the Lancet Countdown project to generate a global indicator to track the trend of direct fire exposure (https://www.lancetcountdown.org/). We used the Collection 6 active fire/thermal anomalies product from the Moderate Resolution Imaging Spectroradiometer (MODIS), which detects fires in 1‐km pixels that are burning at the time of overpass under relatively cloud‐free conditions, to calculate the difference between the mean annual total number of days people were directly exposed to wildfire at the country level during the most recent 4 years (2015–2018), as compared to a 2001–2004 baseline (Giglio et al., 2016). Fire spot locations were matched to country political borders and then joined with gridded population from NASA SEDAC GPWv4 (Watts et al., 2019). The indicator thus is defined as the average annual total person‐days directly exposed to fires in a country. Our wildfire indicator was introduced in the 2019 Lancet Countdown report, filling an important gap in the collaboration's tracking capacity. The results of this indicator were widely covered by major news outlets across the world, with high‐level government and UN political engagement. Going forward, we are working with the Lancet Countdown and stakeholders to further refine and improve this indicator for the 2020 report. This work provides an example of our ability to make the leap from research into practice within a short time frame.

#### 2.1.2 Dust Storm Frequency

Dust contributes more than one third of global aerosol loading (Intergovernmental Panel on Climate Change, 2001). The percentage contributions of dust to surface PM_2.5_ and PM_10_ concentrations are even higher in arid and semiarid regions, which account for ~40% of land on Earth. Health effects associated with dust exposure include increased nonaccidental and cardiovascular mortality, respiratory diseases, and cardiopulmonary diseases (Crooks et al., 2016). Desert dust has also been associated with infectious diseases such as coccidioidomycosis (valley fever) in the southwestern United States (Tong et al., 2017) and meningitis in Saharan Africa (García‐Pando et al., 2014). Approximately 400,000 premature deaths have been attributed to dust exposure annually (Giannadaki et al., 2014). The Indicators Tiger Team adopted a simple indicator, dust storms frequency, to describe the changes in the locations and numbers of dust storms in the United States. Long‐term records of dust storms were reconstructed using a new dust detection algorithm trained with the MODIS true color dust observations (Tong et al., 2012). We found that the frequency of locally originated windblown dust storms has increased 240% in 1990–2011 in the southwestern United States, the same region in which the majority of the valley fever cases were reported (Tong et al., 2017). The dust data are now being used by the Centers for Disease Control and Prevention (CDC) and southwestern states as part of their surveillance of valley fever.

#### 2.1.3 Pollen Counts

Pollen counts are an indicator of exposure that has received less attention than other airborne exposures (e.g., air pollutants), despite the large prevalence of pollen allergy worldwide. As pollen counts are influenced by climate change in several ways (Ziska et al., 2019), the Lancet Countdown was interested in incorporating pollen counts as an indicator. The Tiger Team focused on developing gridded estimates of daily pollen counts for several allergenic pollen taxa in the contiguous United States for the last decade, with the plan to develop similar estimates for other regions of the world pending availability of pollen data for calibrating remotely sensed observations. The pollen count estimates use NASA remotely sensed data on weather and vegetation greenup, or normalized difference vegetation index (NDVI), from MODIS and are expected to be released in the summer of 2020. As the global Lancet Countdown report requires global‐scale exposure estimates, the pollen exposure indicator will not be incorporated into the report until global exposure estimates are available. We are undertaking new efforts to partner with aerobiologists around the world to gather additional pollen data to validate regional models. The Tiger Team advanced methods for the United States and continued building relationships between NASA‐funded investigators and stakeholders to ease the way for a global pollen indicator to be incorporated into the Lancet Countdown in the future.

#### 2.1.4 Urban Green Space Quantity

Green space, including street trees, parks, and gardens, provides multiple climate and health benefits (Frumkin et al., 2017; Hartig et al., 2014; James et al., 2015; Markevych et al., 2017). Green space provides local cooling and promotes physical activity, social engagement, and mental well‐being. The goal of this effort was to create an indicator that would track green space quantity over time in global cities. The MODIS NDVI data used for this project, the same data as for the pollen indicator, have images every 16 days at a 250‐m resolution since 1999 (Carroll et al., 2004). In our initial work, we obtained MODIS data for 467 global urban areas larger than 1 million (obtained from the Global Human Settlement program of the European Commission) quarterly for 2019. Three exposure metrics were calculated for each city: max NDVI (the average greenness value based on the month of maximum exposure); annual average NDVI (updated based on changes in seasonal NDVI); and weighted average greenness values based on population size. Here, the indicator has been developed in response to strong demand from health policymakers and the climate change and health community, both of which are interested in tracking recent and future changes in urban green space (and eventually, the adaptation and health cobenefits of these shifts). Our main stakeholder has been the Lancet Countdown. We expect to include the indicator in the 2020 Lancet Countdown report. We are now in the process of expanding estimation of the indicator from the 467 urban areas to more years and a larger and more representative set of global cities.

### 2.2 Indicators Tracking Air Pollution and Associated Disease Burdens

#### 2.2.1 NO_2_ Concentrations and Asthma Burdens

NO_2_ is a largely urban pollutant that is highly correlated with population and vehicle traffic. NO_2_ differs from ozone and PM_2.5_ in the spatial distribution of its concentrations, relative contributions of its different emission sources, and its effects on health. The GBD Study and State of Global Air reports currently include estimates of exposure to PM_2.5_ and ozone and their attributable disease burdens and have not yet included NO_2_, despite evidence that NO_2_, either alone or as an indicator of a more complex traffic‐related pollutant mix, has been causally associated with various health outcomes, including new onset of asthma among children. Previously, global OMI‐derived NO_2_ concentrations at 0.1° grid resolution were used to generate the first estimates of the global burden of NO_2_ on pediatric asthma incidence (Anenberg et al., 2018). A follow‐on study using a land use regression model for NO_2_ concentrations at 100‐m resolution, with GOME2 and SCIAMACHY satellite observations as inputs (Larkin et al., 2017), more adequately captured high near‐roadway concentrations and led to higher disease burden estimates. An important role of the Tiger Team was to convey to the GBD Study and the State of Global Air the strengths and weaknesses of global NO_2_ concentration estimates generated by integrating satellite observations with chemical transport models or with land use regression models. These discussions identified the importance of incorporating OMI observations into a new land use regression model to generate long‐term NO_2_ concentration trends at high enough spatial resolution to capture near‐roadway concentrations in urban areas. To meet these objectives, the Tiger Team helped launch a new collaboration, sponsored by the Health Effects Institute (which runs the State of Global Air program), between HAQAST researchers and the Institute for Health Metrics and Evaluation (which runs the GBD Study). The resulting new global NO_2_ concentrations are expected to be used to estimate NO_2_‐attributable pediatric asthma burdens globally as a new risk‐outcome pair in the GBD Study.

A new idea that arose during the course of the Tiger Team was to explore whether effects of urban transportation emission reduction policies can be seen from space. In cities, private and public transportation are prominent sources of NO_*x*_ emissions. We employed satellite NO_2_ data from OMI and TROPOMI to analyze tropospheric NO_2_ concentrations over Madrid, Spain, before and after the implementation of the transportation emission reduction policy “Madrid Central.” Early results indicate a reduction in NO_2_ corresponding to the enactment of the regulation in November 2018. Similar analyses may be extended to past and current transportation policy reforms in other cities. This study demonstrates the utility of satellite data for informing air quality, transportation, and public health policy considerations by local governments.

#### 2.2.2 Tropospheric Ozone Concentration and Chemistry

Long‐term exposure to tropospheric ozone is estimated to lead to several hundred thousand premature deaths annually from chronic obstructive pulmonary disease globally (GBD 2017 Risk Factor Collaborators, 2018). Through this Tiger Team, we transferred data and knowledge about ozone concentrations generated by integrating multiple models and in situ measurements. As longer‐term initiatives, we also advanced methods to use satellite remote sensing to constrain modeled ozone concentrations and better understand ozone formation regimes globally. While recent work has demonstrated potential to use satellite remote sensing to retrieve lower tropospheric ozone directly over regions where boundary layer concentrations are sufficiently high (Shen et al., 2019), this approach does not work over most of the globe.

Prior to the GBD 2017 study (GBD 2017 Risk Factor Collaborators, 2018), GBD studies based global ozone concentrations on output from a single global atmospheric model (Brauer et al., 2016). With HAQAST support, we improved estimates of global ozone concentrations by statistically fusing surface ozone observations with output from multiple global atmospheric models, to support the GBD 2017 and GBD 2019 studies. We used ozone observations from the Tropospheric Ozone Assessment Report (TOAR), the largest compilation of global ozone observations to date (Schultz et al., 2017), as well as several atmospheric models, including several from the Chemistry Climate Model Initiative (Morgenstern et al., 2017). Whereas previous studies have often used multimodel averages for similar efforts, for GBD 2017, we developed methods of combining multiple atmospheric models based on their performance in matching observations in several world regions (Chang et al., 2019. These methods were then improved for the forthcoming GBD 2019. We used new ozone observations including from China and more model results that extended beyond 2010. We also used the Bayesian Maximum Entropy method (Christakos et al., 2001) to smoothly integrate observations and the multimodel composite of simulated ozone concentrations in both space and time. Finally, we added fine spatial structure to our estimations by scaling relative to a fine‐resolution global model simulation (Hu et al., 2018). We delivered ozone concentrations at 0.1° resolution for each year from 1990 to 2017 for the GBD 2019. This data set is available for other uses, such as for risk assessments or epidemiologic studies of ozone health effects.

Through the Tiger Team, we also worked to improve estimates of ozone concentrations and trends by assimilating satellite observations globally. Satellite data can mitigate shortcomings in model ozone estimates, which may lack up‐to‐date emissions information in all areas of the world, and help fill in gaps in ozone monitoring data, especially outside of the United States, Europe, and China. A global atmospheric chemical transport model (GEOS‐Chem) was merged with satellite NO_2_ observations (including both NASA v3 and DOMINOv2 products) to constrain emissions of NO_*x*_, a key precursor of ozone from 2005 to 2016 (Qu et al., 2020). The 4D‐Var approach was used with the GEOS‐Chem adjoint model to make adjustments to monthly NO_*x*_ emissions in each 2° × 2.5° grid cell globally. Compared to surface measurements, simulations based on assimilation of the NASA product lead to smaller bias and error for all ozone metrics examined. This activity remains under development and in the future can improve surface ozone concentration estimates for the GBD.

In addition to improving ozone concentrations, the Tiger Team used satellite observations to advance understanding of ozone chemistry regimes globally, which has implications for designing policy measures for ozone abatement. Ozone is produced from photochemical reactions involving its precursors: nitrogen oxides (NO_*x*_) and volatile organic compounds (VOCs). For over two decades, satellite instruments have provided continuous global observations of tropospheric column NO_2_ and formaldehyde (HCHO), which can serve as indicators for NO_*x*_ and VOCs, respectively. The satellite‐based HCHO to NO_2_ ratio has been used to infer whether ozone formation is limited by NO_*x*_ or VOCs or in transition between regimes (Duncan et al., 2010; Jin & Holloway, 2015; Martin et al., 2004). Our ongoing research demonstrates that satellite‐based HCHO/NO_2_ generally captures the observed nonlinear dependence of ozone production on NO_*x*_ and VOCs (Jin et al., 2020). In addition, observed decadal changes in spatiotemporal patterns of ground‐level ozone, known to occur as ozone formation chemistry transitions to stronger NO_*x*_ sensitivity, are linked directly to satellite HCHO/NO_2_ trends over several U.S. metropolitan areas. This recent work provides added evidence that ozone formation has become more sensitive to NO_*x*_ emissions over major cities such as New York, Chicago, London, Beijing, and Seoul, as NO_*x*_ emission controls have been implemented (Jin et al., 2017). These results imply that NO_*x*_ emission controls will reduce ozone more now than they would have in the past. Thus far, we have worked with stakeholders to develop a technical document on using satellite‐based HCHO/NO_2_ for State Implementation Plans in the United States (Jin et al., 2018) and are exploring with other stakeholders how this indicator can inform urban ozone abatement strategies.

#### 2.2.3 Urban PM_2.5_ Mortality Burdens

Urban PM_2.5_ mortality burdens are an example of an indicator where methods and data existed prior to the Tiger Team, and a focused effort was needed to repackage the data to be more directly useful for stakeholders. The GBD has included PM_2.5_‐attributable mortality burdens for many years, but they are primarily provided at national or at most, subnational (e.g., state, province, or county) scales (Cohen et al., 2017; GBD 2017 Risk Factor Collaborators, 2018; World Health Organization, 2016b). City‐level PM_2.5_ mortality burden estimates are increasingly of interest, as populations are growing in many cities where air quality is also worsening (United Nations, 2014; World Health Organization, 2016a). We therefore developed the first estimates of urban PM_2.5_ mortality burdens for 250 cities worldwide, using methods that were consistent globally and compatible with the GBD Study (Anenberg et al., 2019). Our estimates were enabled by years of research that preceded our study to generate high‐quality gridded (0.1° × 0.1°) estimates of surface PM_2.5_ concentrations globally by integrating AOD observations from multiple satellites with global chemical transport models and ground observations (Shaddick et al., 2018; van Donkelaar et al., 2016). We used these concentration estimates to generate PM_2.5_‐attributable mortality burdens at the same 0.1° × 0.1° resolution and then aggregated the gridded burdens within city boundaries. We made these urban PM_2.5_ mortality burdens available publicly and shared them directly with various stakeholders. C40 Cities are building a globally consistent scoping tool for local‐scale climate action planning, and as part of the tool, the urban PM_2.5_ disease burdens fill a critical need for baseline air pollution levels and associated disease burdens. C40 Cities also used our urban PM_2.5_ mortality burdens to estimate the benefits of meeting World Health Organization Air Quality Guidelines across many cities (C40 Cities, 2019). By highlighting air pollution health impacts in cities, these stakeholders are working to motivate city‐level actions to jointly mitigate climate change and air pollution.

## 3 Discussion

The NASA HAQAST Indicators Tiger Team facilitated the transfer of global‐scale satellite remote sensing and related data to stakeholders for tracking the world's progress toward mitigating air pollution and climate change. By the end of the year‐long Tiger Team, population wildfire exposure had been incorporated into the latest Lancet Countdown report (Watts et al., 2019), the GBD Study and the State of Global Air are incorporating the new ozone concentrations (Chang et al., 2019; GBD 2017 Risk Factor Collaborators, 2018) and are considering integrating the new NO_2_ concentrations and asthma burdens (Achakulwisut et al., 2019), and the Climate and Clean Air Coalition and C40 Cities are using urban PM_2.5_ mortality burdens (Anenberg et al., 2019) to inform city planning for air pollution and climate change mitigation. The Lancet Countdown is also incorporating our wildfire and green space estimates in future iterations. The project helped facilitate the development of new methods for estimating pollen exposure worldwide, dust storm frequency, and global ozone concentrations (Chang et al., 2019; Qu et al., 2020). In the coming years, we anticipate seeing ripple effects of these outputs in both end‐user efforts and scientific studies.

The Tiger Team also advanced the field in less tangible yet highly influential ways. We accelerated capacity building among the stakeholders to understand and utilize satellite remote sensing products, helping to broaden the acceptability of satellite remote sensing measurements for global and regional air quality management and public health applications. We also forged new and strengthened existing relationships between NASA‐funded scientists and end users of remotely sensed data and generated novel applied research ideas that were informed by new understanding of stakeholder needs. These advances will influence our activities for years to come, as we further develop the foundation‐building work we started through the Tiger Team to spin off new collaborative efforts that address emerging stakeholder needs and leverage ever‐improving satellite products. They will also inform mitigation efforts at multiple scales, including by intergovernmental organizations, national governments, and municipal governments.

Several lessons learned from our experience may be helpful for future science‐stakeholder collaboratives engaging in convergence research. First, building understanding between the researcher capabilities and stakeholder needs takes time and effort. Multiple teleconferences and discussions at in‐person meetings were needed to build mutual understanding and relationships between the researchers and stakeholders. These relationship‐building exchanges included smaller spin‐off discussions on individual topics, where stakeholders and scientists could engage in two‐way communication to develop a shared understanding of needs and capabilities. In some cases, it was more effective for the researchers to engage directly in stakeholder processes, for example, participating in teleconferences and in‐person meetings with established working groups. This messy, heterogeneously organized process of coproducing knowledge with stakeholders has been documented in the literature on actionable science and its application to other societal problems (Kirchhoff et al., 2013; Norström et al., 2020). Our experience provides further evidence that participatory knowledge production can lead to more relevant, actionable, and useful information for stakeholders but requires the devotion of time and attention from both scientific researchers and stakeholders to be effective.

Second, flexibility is a critical aspect of the structure of HAQAST and its Tiger Teams. New priorities arise as stakeholder needs evolve and new synergies emerge when there are engagements across a wide range of stakeholders and teams. The organization of HAQAST around responding quickly to stakeholder needs enables researchers to pivot and form innovative smaller subset teams of individuals with the expertise needed to address the problem. This was the case for NO_2_ and asthma; with stakeholder interest in incorporating NO_2_ and asthma as a new risk‐outcome pair in the GBD and State of Global Air reports, we were able to quickly assemble a team with diverse expertise who could generate and interpret data.

Third, our efforts to transfer mature data sets to end users, to further develop more nascent indicators, and to generate ideas for future indicators in parallel allowed us to advance our mission in the near term while also putting in place the building blocks for new indicators to be included in the long term. Engaging with stakeholders is valuable at each stage of data development to ensure that methods and results are compatible with user needs.

Fourth, sustained collaboration between the researchers and stakeholders after the extramural support ends remains a challenge. Several stakeholders follow an iterative process with reports published annually and continue to rely on external scientists to contribute effort and expertise toward providing and interpreting updated data sets. Funding for these annual reports supports the synthesis required for their production, but not the data collection and analysis required for exposure assessment. Since researchers are not typically supported by grant funding to do this type of work or professionally evaluated by the amount of “research translation” work they do, the HAQAST structure has accelerated the integration of cutting‐edge science into stakeholder activities in ways that may have been otherwise unlikely to occur. Identifying ways to support researcher‐stakeholder collaborations in a sustained way could unlock the potential for researchers to meet stakeholder needs on longer time frames and support the development of the GBD, Lancet Countdown, and State of Global Air, and other stakeholder reports as public goods.

Fifth, there is an important interplay between remotely sensed data and the ground monitoring data necessary to “ground truth” or calibrate satellite‐based estimates. Ground measurements are needed to allow us to take advantage of remotely sensed variables and use these observations to generate global estimates of environmental exposures and their associations with health outcomes. For exposures such as pollen, for which remote observations are important but insufficient to generate exposure estimates, we may need to wait on additional ground‐based observational networks to fully realize the potential of satellite observations.

Finally, the Indicators Tiger Team demonstrated the multiple benefits from global‐scale applied research sponsored by NASA. First, this collaboration will support international efforts to mitigate air pollution and climate change, which span national boundaries and require intergovernmental action to address. By producing research at the global scale and working with international teams to track air pollution and climate change indicators, we help move nations further toward reducing emissions, which will benefit public health both globally and in the United States. Working with and learning from international teams also improves methods and tools developed in the United States, which can be applied to support reducing U.S. and international emissions. Beyond advances to scientific methods and tools, building relationships and collaborations that span national boundaries further enhances U.S. investigator development and expertise. There are thus many ways that U.S. government sponsorship of global‐scale research can benefit the United States, including for improving U.S. public health and for promoting the excellence of U.S. researchers and work products. These benefits can also be achieved by support for global‐scale air quality and climate change research from other U.S. grant funding agencies.

Satellite remote sensing has transformed our ability to track the world's progress in mitigating global air pollution and climate change (Cromar et al., 2019; Duncan et al., 2016; Krotkov et al., 2016). The researcher‐stakeholder collaborations supported by NASA HAQAST and its Indicators Tiger Team have accelerated the transfer of NASA science from a variety of satellite sensors (OMI, MODIS, etc.) into high‐impact stakeholder activities. Upcoming launches of pollution‐observing satellites will further advance our ability to track progress on air pollution and climate change, particularly in individual countries using geostationary satellites (e.g., TEMPO) and in megacities globally (MAIA). With these satellite sensors, along with other advances in existing and new technologies (e.g., chemical transport modeling, low‐cost sensors, and mobile monitoring), we are entering a new age of global, highly spatially resolved, and long‐term data availability that provides an unprecedented ability to understand and track planetary‐scale environmental changes. As these new capabilities will still require individuals and teams to build bridges from the scientific observations into operational data sets and knowledge, we hope the experience of the HAQAST Indicators Tiger Team can inform future problem‐driven science‐stakeholder collaborations.

## Conflict of Interest

The authors declare that they have no conflicts of interest relevant to this study.

## Data Availability

Data sets for each indicator will be shared publicly upon publication of complementary technical journal articles describing the methods for each one.
